# Laser-Induced Graphene Interfaces with Controlled Electrical Conductivity, Topography and Wettability for Biomedical Applications

**DOI:** 10.1021/acsanm.5c05398

**Published:** 2025-12-16

**Authors:** Lidia Lizbeth Hernández-Cubas, Paola Sánchez-Moreno, Andrea Capasso, Modesto T. Lopez-Lopez, Alejandro Moltó-Ramírez, Noel Rodríguez, Mattia Bramini, Carmen Lucía Moraila-Martínez

**Affiliations:** † Departamento de Biología Celular, 16741 Universidad de Granada, E-18071 Granada, Spain; ‡ Departamento de Electrónica y Tecnología de Computadores, Universidad de Granada, E-18071 Granada, Spain; § Facultad de Biología, Universidad Autónoma de Sinaloa, MX 80000 Culiacán Rosales, Sinaloa, Mexico; ∥ Departamento de Física Aplicada, Universidad de Granada, E-18071 Granada, Spain; ⊥ 246702 International Iberian Nanotechnology Laboratory, PT 4715-330 Braga, Portugal; # Instituto de Investigación Biosanitaria Ibs.GRANADA, E-18012 Granada, Spain; ¶ Universidad de Granada, Research Unit “Modeling Nature” (MNat), C.U. Fuentenueva, E-18071 Granada, Spain

**Keywords:** laser-induced graphene, conductivity, topography, wettability, scaffolds, biological applications, biocompatibility

## Abstract

Graphene-based materials hold great potential for the development of neural interfaces; however, conventional fabrication techniques often involve costly and intricate processes, limiting their scalability and practical implementation. In contrast, laser-induced graphene (LIG) provides a highly scalable, cost-effective, and direct laser-writing technique for the fabrication of nanostructured graphene-like sheets. LIG enables the rapid and accessible production of customizable substrates without the need for complex processing or expensive precursors. Moreover, its versatility allows for precise control over laser parameters, allowing the fine-tuning of critical physicochemical properties such as electrical conductivity, wettability, and surface roughness. This adaptability makes LIG an attractive platform for engineering graphene-based biomaterials, particularly for neural interfaces, where surface characteristics influence key biological responses, including cell adhesion, proliferation, and differentiation. In this study, we engineered and characterized three distinct LIG substrates with tailored topographies, defined patterns, and controlled physicochemical properties, assessing their stability under biological environments. Systematic analysis of wettability, surface roughness, mechanical and electrical properties revealed that these parameters remain stable under physiological conditions. Furthermore, preliminary biocompatibility assays using neural-like cells demonstrate encouraging results. Notably variations in laser-induced patterning significantly influenced cellular behavior, with specific topographies enhancing adhesion and promoting guided cellular alignment. These findings highlight the critical role of surface architecture in modulating cell responses, reinforcing the potential of these substrates for neuro-biomedical applications. Our work highlights the potential of LIG as a tunable and scalable strategy for the development of next-generation neural interfaces and pave the way for future studies aimed at harnessing LIG’s versatility for next-generation neural interfaces.

## Graphical abstract

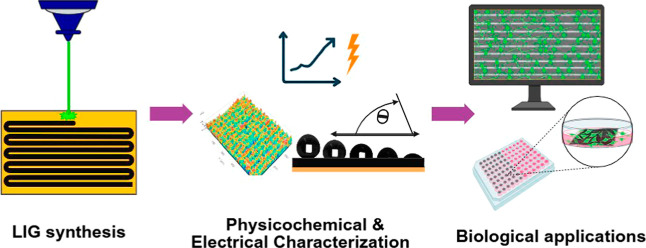

## Introduction

Ongoing scientific and technological advancements have driven the development of novel materials to address emerging needs in fields such as flexible electronics, photovoltaics, industry, and biomedicine. Among these materials, graphene has attracted significant research attention due to its exceptional properties, including high electrical conductivity, flexibility, transparency, mechanical strength, and low density.^1^ Biomedical applications at large and tissue engineering in particular, require biocompatible and flexible polymer-based implants to be adjusted to the specific needs of the patient to perfectly match and address the tissue lesion. In the neurobiology field, having a flexible and conductive substrate able to promote cellular adhesion and growth, together with electrical stimulation, would have a crucial impact to restore the neuro-signaling loss-of-function due to a lesion.^2,3^ This is a crucial aspect in the case of the central nervous system (CNS), which has a limited capacity for self-repair and regeneration and is highly prone to neurodegeneration. Thus, adjusting the surface properties of substrates, such as increasing wettability, electrical conductivity, and topography, to the specific biocharacteristics of the lesion, will be the path to pursue in the future neural-tissue engineering field. In this context, graphene-based materials (GBMs) have already proved to exhibit significant potential for interacting with various cell types, facilitating tissue repair and reconnection.^4–8^ However, there are several parameters that all combined play a key role in this application. Among all, it has been shown that electrical stimulation enhances neurite outgrowth^9^ and surfaces with rough/patterned topography, as well as high wettability, have proven to promote stronger cell adhesion.^10^ Recent work has highlighted the potential of graphene and graphene-based coatings as neural interfaces, but graphene also presents some limitations and new approaches are emerging.^11–13^ Electrical conductivity, while not the fundamental parameter for excitable neural networks,^14^ is still highly recommendable for neuronal cells. In addition to conductivity, the surface chemical properties, such as wettability, and structural properties, like thickness and roughness, play a pivotal role in promoting cell adhesion and maturation.^15,16^ At the device level, graphene-based biomedical interfaces exploit a combination of physical properties: (i) high charge carrier mobility and low interfacial impedance for efficient electrical recording and stimulation; (ii) mechanical flexibility and low thickness for conformal contact with soft tissues; and (iii) large specific surface area for enhanced charge transfer and biological performance. The latest includes overall biocompatibility, support of neurite outgrowth and synaptic activity, and the ability to modulate cell adhesion through tailored surface chemistry and topography.^11,17^ Large-scale synthesis of graphene is complex and expensive, and in case of chemical-vapor deposition (CVD) requires a substrate (usually copper) that needs to be removed after the process, adding issues related to potential mechanical damage and contamination.^18^ Graphenés surface is inherently hydrophobic and flat, and it can thus limit specific biological events, such as cell adhesion, sprouting, and division. Furthermore, precise and clean patterning of an atomic-thick surface is usually challenging. These limitations have driven research toward graphene-based materials (GBMs) that retain the advantageous electrical and mechanical properties of graphene while offering more favorable surface chemistry and topography for biological interactions. These materials can be synthesized using various techniques, such as graphene oxide (GO), reduced graphene oxide (rGO), and laser-induced graphene (LIG), which all require a specific production technique. The latter technique allows the large-scale production of nanostructured graphene-like sheets through laser radiation proving to be a simpler and more cost-effective method.^19^

LIG has attracted growing interest in biomedical research.^20–22^ Its fabrication process relies on laser photoablation of carbon-rich polymers such as polyimides is a direct route to obtain nanostructured graphene sheets with controlled properties.^23^ This process breaks down the carbon bonds forming the polyimide structure via various mechanisms, such as the excitation of charged particles, ultraviolet (UV) laser radiation, pyrolysis processes, or thermal treatments.^24^ The energy emitted by the laser efficiently removes oxygen-containing functional groups from the raw material, as well as most C–N, C–O–covalent functionalization with bioactive molecules in neural interface applications.

C, and C=O bonds present in the polyimide structure.^25^ This results in a valuable combination of physical and chemical characteristics, including high electrical conductivity (resembling that of conventional graphene substrates) and, at the same time, surface roughness, wettability, and the ability to undergo chemical functionalization, which enhances biological interactions with living systems.^26–28^ Finally, the surface topography and wetting properties of LIG films can be designed and tailored by specific laser photoablation protocols to induce desired physicochemical modifications (custom patterning and roughness), pushing forward the research on the topic. Thus, the unique properties of LIG might support cell adhesion, growth, and cell stimulation, key factors for the development of effective future neuro-interfaces and neuro-prosthetics.

The interplay between micro/nanostructured roughness and surface chemistry in GBMs is emerging as a key design parameter for advanced biointerfaces. Recent studies in smart medical systems have shown that engineered GBM architectures and controllable functionalization can be used to modulate protein adsorption, cell adhesion and electrical coupling in flexible or implantable platforms.^29,30^ In line with these advances, the micro/nanostructured roughness of LIG and its moderate density of oxygen-containing groups (e.g., C–O, C=O and O–C=O) are expected to govern bio interfacial interactions and provide chemically addressable sites for covalent functionalization with bioactive molecules in neural interface applications.^31,32^

This work shows a reliable and scalable approach to produce LIG as a promising biocompatible scaffold in alternative to CVD-graphene-based supports in applications where direct patterning, surface roughness and cost-effective scalability are critical. LIG was produced by laser irradiation on carbon-rich Kapton polyimide substrates.^13^ By adjusting the etching parameters and designing different patterns, LIG substrates can be tailored to specific applications, optimizing their conductivity, roughness and wettability, and expanding their potential in biomedical and other high-tech fields. Specifically, we fabricated three LIG substrates that differ in their topography and conductivity. For all LIG substrates, an extensive electrical, structural, mechanical, and physicochemical characterization, including morphology and wettability, was conducted. Subsequently, preliminary biological assays were explored to assess the in vitro biocompatibility of LIG. Our results demonstrate the ability to obtain diverse materials with optimal properties for biological environments in terms of conductivity, roughness, and wettability. This was achieved by varying laser power/speed configurations and pattern design. Recent studies (Table S1) have further demonstrated the potential of LIG and other GBMs in biosensing and cell-scaffold applications, often focusing on device performance or specific cell responses.^33–35^ However, many of these approaches rely on additional coatings or complex postprocessing to optimize wetting and cell adhesion or provide limited control over surface patterning and mechanical behavior. In contrast, the present work offers a single-step, scalable strategy to fabricate patterned LIG on flexible Kapton that combines low sheet resistance, a high-adhesion wetting regime without extra functionalization, preserved tensile flexibility and direct evidence of cell viability and contact guidance on the as-fabricated substrates, thereby integrating electrical, mechanical and interfacial characterization within one platform. The exceptional flexibility of LIG films in customizing diverse properties not only provides a valuable platform for engineering materials with controlled surface and structural characteristics, highlighting their immense potential in biomedical applications, but also holds significant promise for advancing new materials, electronics, and the biotech industry due to the ease of scaling up the LIG fabrication process.

## Experimental Section

### Kapton Laser Induction Process

The laser-induced graphene (LIG) synthesis was performed using a Coherent Power Line E-12-532 pulsed laser system (Munich, Germany), a galvanometric pulsed laser with a wavelength of 532 nm and a maximum power density of 6 kW/cm^2^.^36^ To ensure stable and reproducible processing conditions, the laser was prestabilized by operating at 0.75 W for 30 min before initiating the engraving process. The patterning was conducted under ambient conditions, utilizing a multipulse scanning strategy in which the laser traced overlapping horizontal lines, following preprogrammed designs in Coherent’s Visual Laser Studio software. For this study, three distinct laser-patterned designs were generated, denoted as LIG_A, LIG_B, and LIG_C (Figure 1a). LIG_A consists of parallel lines, LIG_B of an orthogonal grid (two passes at 90°) and LIG_C of an oblique cross-hatched pattern (two passes at an angle ≠ 90°) with the same line spacing. Each 10 × 10 mm sample was fabricated and subsequently prepared for comprehensive morphological, structural, and physicochemical characterization. To assess process reproducibility, four independent batches were produced for each pattern type, ensuring statistical robustness in subsequent analyses. The critical fabrication parameters used for each design, including laser power, scanning speed, repetition frequency, line width, line spacing, number of passes, laser spot diameter and the corresponding power density (in kW/cm^2^), are summarized in Table 1 to facilitate reproducibility.

**Figure 1:**
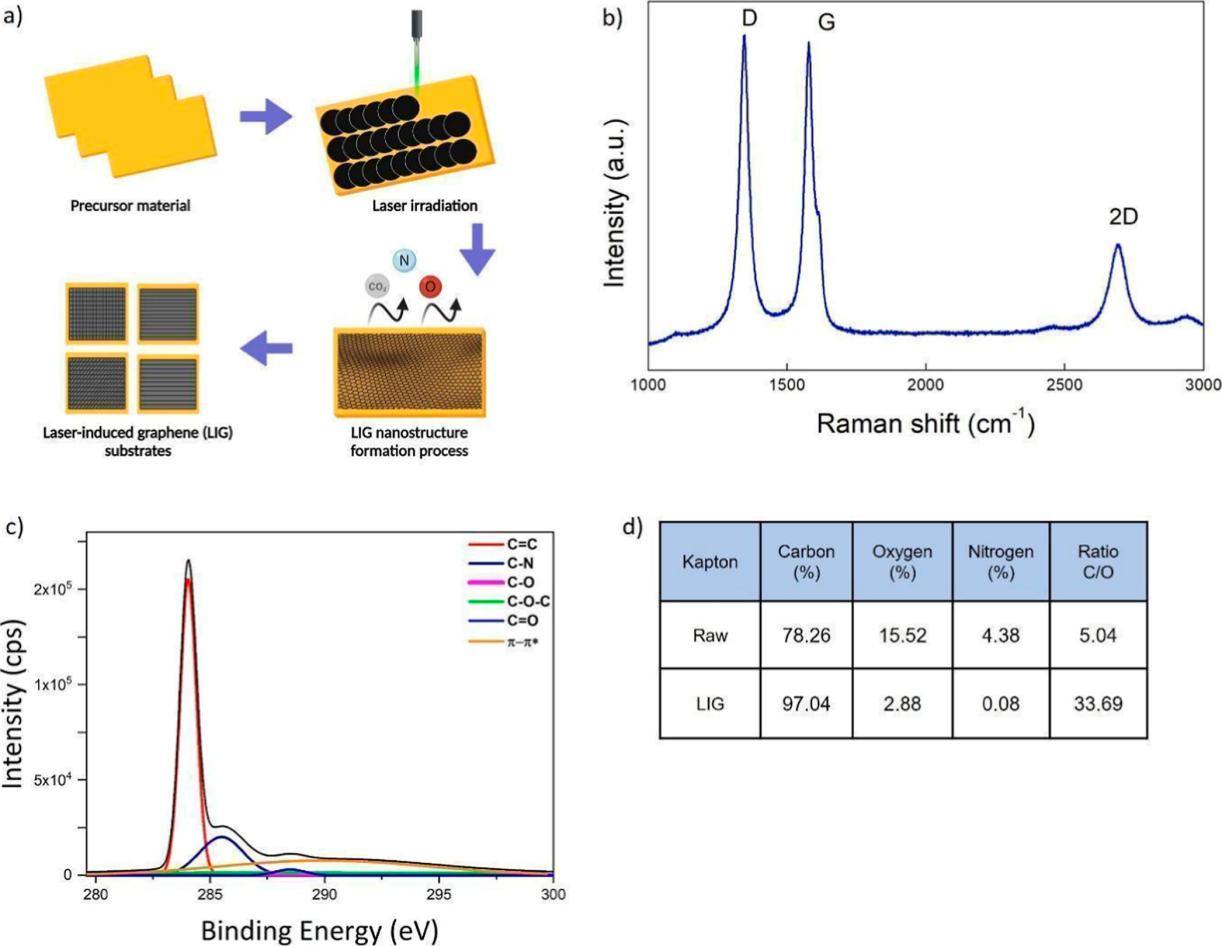
LIG substrate synthesis and their morphological structures. (a) The schematic diagram illustrates the LIG synthesis. (b) Raman spectrum of LIG substrates (representative of LIG_A, LIG_B and LIG_C). (c) High-resolution XPS of LIG substrates. (d) Atomic percentages of carbon, nitrogen, and oxygen in Kapton polyimide before and after laser treatment performed by Kratos Axis Ultra-DLD X-ray Photoelectron Spectrometer.

**Table 1:** Laser Fabrication Parameters and Pattern Geometry for LIG_A, LIG_B and LIG_C

parameters	LIG_A	LIG_B	LIG_C
laser power (W)	1	1	1
scanning speed (mm/s)	60	60	60
frequency (kHz)	50	50	50
line width (μm)	50	50	50
line spacing (μm)	50	50	50
number of passes	1	2	2
spot diameter at the sample plane (μm)	35.2	35.2	35.2
power density (kW/cm2)	6	6	6

### Electrical Characterization

The electrical properties of the LIG substrates were evaluated using a Keysight B2902A Source Meter Unit (SMU) (Santa Rosa, CA, USA) to measure voltage and current across the different laser-patterned designs. Sheet resistance measurements were performed using a Jandel Universal Probe Station (Eggington, UK) equipped with an In-Line Four-Point Probe configuration, with 1 mm spacing between needles. The dual-configuration setup was employed to enhance measurement precision and minimize contact resistance effects.^31^ Each LIG substrate, fabricated under specific laser power and scanning speed conditions, was subjected to 30 individual measurements per batch under ambient conditions. To ensure statistical robustness, the procedure was repeated across three independent replicates per batch. The final resistance value was calculated as the means of all recorded measurements, providing a comprehensive assessment of the electrical performance of the different LIG architectures.

### Evaluation of Electrical Performance of LIG Substrates in Biological Media

To assess the stability and performance of the electrical properties of LIG substrates in biological environments, resistivity measures were performed before and after exposure to different biological media. These measurements provide key insights into potential variations in conductivity that may arise due to interactions with physiological conditions. The experimental setup for electrical resistance measurements is detailed in Figure S1. The resistivity characterization was conducted using Keysight Technologies Inc. B2912A Precision Source Measurement Unit (SMU), controlled via Quick IV software (Keysight, Santa Rosa, CA, USA). Current–voltage (*I*–*V*) curves were recorded, and electrical resistance was determined from the inverse of the slope of the linear region, following Ohm’s law. Triplicate measurements were carried out on three different substrates to ensure consistency.

### Structural and Chemical Characterization of LIG Substrates

The structural and chemical properties of LIG substrates were analyzed using Raman spectroscopy and X-ray Photoelectron Spectroscopy (XPS) to assess the graphitization degree, defect density, and surface chemical composition. Raman spectra were acquired using a JASCO NRS-5100 microdispersive Raman spectrometer (Easton, PA, USA), equipped with a 532 nm green diode laser (Elforlight G4–30; Nd:YAG). Spectral acquisition was performed using 10 accumulations with a 3 s exposure time per scan to enhance the signal-to-noise ratio (SNR). To further investigate the chemical composition and bonding states of the LIG surface, X-ray Photoelectron Spectroscopy (XPS) was conducted using a Kratos Axis Ultra-DLD spectrometer (Manchester, UK). The measurements were performed with an Al Kα X-ray source (*hv* = 1486.6 eV) operating at 450 W in an ultrahigh vacuum (UHV) chamber, where the pressure was maintained below 10^–10^ Torr.

### Surface Morphology and Roughness Analysis of LIG Substrates

The morphological features and roughness of the LIG substrates were analyzed using 3D topography reconstruction obtained via white-light confocal microscopy (PLμ, Sensofar Tech S.L.). The system was equipped with 10× (1.39 × 1.02 mm^2^) and 20× (695.5 × 509.18 μm^2^) objectives to achieve varying levels of spatial resolution. To evaluate surface roughness, the microscope scanned the area of interest, generating high-resolution topographies based on transmitted light intensity variations. The thickness of each scan plane and the scanning area were determined based on the selected microscope objective and scanning speed. The quantitative roughness parameters, including mean roughness (Ra), root-mean-square roughness (Rq), and peak-to-valley distance (PV), were calculated using Gwyddion software (an open-source topographic analysis tool). The reported roughness values correspond to Rq measurements within a defined area of the substrate surface. To ensure statistical robustness, five independent regions were analyzed per substrate, with measurements conducted across three separate samples for each LIG pattern.

### Wettability Measurements and Surface Interaction of LIG Substrates

To evaluate the suitability of LIG substrates for biological applications, we characterized their surface wettability, a critical factor that directly influences media absorption and cell adhesion. Wettability was assessed through contact angle hysteresis measurements, which provide a dynamic description of how liquids interact with the substrate surface. This includes both advancing and receding contact angles, following the methodology described by Moraila-Martínez et al.^37^ Measurements were performed using a PSD3 microinjector (Hamilton), coupled with a 250 μL Hamilton syringe, which allowed for precise and reproducible liquid injection and retraction. A 20 μL droplet of distilled water was initially deposited to form a centered and expanding droplet, with the maximum drop volume set at 200 μL to ensure sufficient spreading and retraction range. For data acquisition and analysis, we employed the Dinaten and Contacto software, developed by Dr. Juan Antonio Holgado Terriza at the University of Granada. The drop profiles were analyzed using the Axisymmetric Drop Shape Analysis-Profile (ADSA-P) technique, enabling extraction of parameters such as advancing and receding contact angles, contact radius, droplet area, volume, and surface tension. This methodology allowed us to detect even subtle changes in surface hydrophilicity induced by different laser-patterned designs, providing critical information about the biointerface readiness of the LIG substrates for use in cell culture and tissue engineering.

### Mechanical Characterization of Kapton and LIG Substrates

To characterize the mechanical performance of the laser-induced graphene (LIG) interfaces, tensile tests were performed. Measurements were carried out on a Discovery HR-1 rheometer (TA Instruments) equipped with a tensile geometry comprising two clamps. LIG patterned samples were cut into rectangular strips, ensuring an exposed gauge length of 12 mm between the clamps. The width and thickness of each sample were measured prior to testing using a vernier calliper. Experiments were conducted at a strain rate of 1% per second (corresponding to 120 μm/s), while the tensile force was continuously recorded and converted to tensile stress. In all cases, a small prestrain between 0 and 60 μm was applied before the measurement to remove any slack or creases in the samples; the force during this prestrain was kept below 1 N to avoid underloading or overloading that could compromise the accuracy of the Young’s modulus determination. In addition to the LIG-patterned samples, untreated Kapton films were tested as controls to assess the impact of the laser treatment on the mechanical properties of the polyimide. At least five measurements were performed for each sample type to ensure statistical reliability, and the standard deviation was used to estimate the experimental error.

### Preparation of Substrates for Cell Culture

For cell culture, in addition to LIG substrates, glass and untreated Kapton substrates were used as controls. The substrates were sterilized by sequentially rinsing three times with 70% ethanol (EtOH) followed by three rinses with sterile water. Subsequently, the substrates were transferred to a 12-well plate and allowed to dry under aseptic conditions at room temperature for 3 h. All procedures were conducted in a laminar flow hood (Telstar Bio-II-A).

### Cell Culture

U87-MG human glioblastoma cell line and N2A neuroblastoma cells were obtained from the Centre of Scientific Research (CIC, UGR). U87-MG cells were cultured in Dulbecco’s Modified Eagle Medium (DMEM; PAN Biotech, Germany), supplemented with 10% (v/v) fetal bovine serum (FBS, Gibco, MA, USA) and 1% penicillin–streptomycin solution (PS; Gibco, MA, USA). The cell culture was maintained in an incubator (Biotech) at 37 °C in a humidified atmosphere of 5% CO_2_ and 95% humidity. The medium was replaced every 2 days. N2A neuroblastoma cells were maintained in DMEM/Ham’s F-12 supplemented with 10% fetal calf serum (FCS), penicillin (100 U/mL), streptomycin (100 U/mL), 1 mM l-glutamine and 1 mM sodium pyruvate. Cells were thawed following standard procedures, expanded in T75 flasks and cultured at 37 °C in a humidified incubator with 5% CO_2_ until reaching 70–80% confluence.

### U87-MG Fluorescence Staining and Imaging Acquisition

The U87-MG cells grown onto LIG substrates for 48 and 96 h were fixed with 4% paraformaldehyde (PFA, Sigma-Aldrich, MO, USA) in PBS for 15 min at RT. To prevent nonspecific antibody binding and reduce background noise, a blocking solution of 2% bovine serum albumin (BSA, Slgma-Aldrich, MO, USA) in PBS was applied for 30 min at RT. Cell nuclei were stained with Hoechst 33342 (1 μm, Thermo Fischer Scientific, MA, USA), while Alexa Fluor-647 Phalloidin (Invitrogen) was used to label the F-actin cytoskeleton. Finally, the substrates were mounted on glass slides with Mowiol (Sigma-Aldrich, MO, USA) and left dry overnight at RT. Fluorescence imaging was performed using a Leica DMI RB inverted fluorescence microscope (Leica Microsystems, Germany) equipped with a Leica K5 camera and a 20× objective. Five fields of view were acquired per sample. Images were analyzed with FIJI ImageJ software.

### N2a Growth and Differentiation Media

N2a growth medium consisted of DMEM/Ham’s F-12 (GlutaMAX) containing 1% nonessential amino acids (NEAA), penicillin (50 U/mL), streptomycin (50 μg/mL) and 10% fetal bovine serum (FBS). The differentiation medium used the same basal formulation with 2% FBS and was supplemented with albumin (1 mL, 7.5%) and retinoic acid (100 μL, 10 mM).

### N2a Substrate Preparation

Glass coverslips were coated with 0.2–2% gelatin. Kapton and LIG substrates (LIG_A, LIG_B, LIG_C), together with glass controls, were sterilized by immersion in 70% ethanol followed by UV exposure and then placed in 12-well plates.

### N2a Cell Seeding and Differentiation

N2a cells were seeded at 4 × 10^4^ cells per well onto LIG substrates, glass controls and Kapton. After 24 h in the growth medium, the medium was replaced with differentiation medium, and cultures were maintained for 48 or 96 h prior to imaging and biochemical analysis.

### N2a Cell Viability and Density Quantification

Cell viability and death were assessed by fluorescence staining using fluorescein diacetate (FDA), Hoechst-33342 and propidium iodide (PI). FDA labels viable cells through intracellular esterase-mediated cleavage, Hoechst stains all nuclei and PI marks cells with compromised membranes. Stock solutions were prepared as follows: FDA (5 mg/mL in acetone, stored at −20 °C), Hoechst 33,342 (1 mg/mL in DMSO, −20 °C) and PI (1 mg/mL in Locke’s solution, 4 °C). A fresh working solution was prepared in Locke’s buffer containing FDA (15 μg/mL) and PI (5 μg/mL) in a final volume of 10 mL. Cells grown on the different substrates (glass, Kapton and LIG patterns) were incubated with ∼1 mL of staining solution for 3 min at room temperature. After incubation, cells were washed with Locke’s solution and immediately imaged using a Leica DMI RB inverted fluorescence microscope equipped with a Leica K5 camera and a 20× objective. Five random fields per sample were acquired and analyzed using FIJI (ImageJ). Cell density was quantified by counting Hoechst-stained nuclei (cells/mm^2^). Viability was calculated as the percentage of FDA-positive cells relative to total nuclei, and cell death as the percentage of PI-positive nuclei. These parameters were evaluated for each substrate at 48 and 96 h. Results are expressed as mean ± SD from at least three independent experiments.

## Results

Laser-induced graphene (LIG) patterns were fabricated directly on Kapton polyimide substrates by laser irradiation, yielding three configurations denoted as LIG_A, LIG_B and LIG_C. LIG_A consists of parallel laser-written lines, LIG_B of an orthogonal grid generated by two passes at 90°, and LIG_C of an oblique cross-hatched pattern produced by two passes at an angle different from 90°. The detailed laser processing parameters and pattern geometries for each configuration are summarized in Table 1 in the Experimental section, and all characterizations shown in Figures 1–4 refer to LIG substrates prepared using this fabrication protocol.

**Figure 2:**
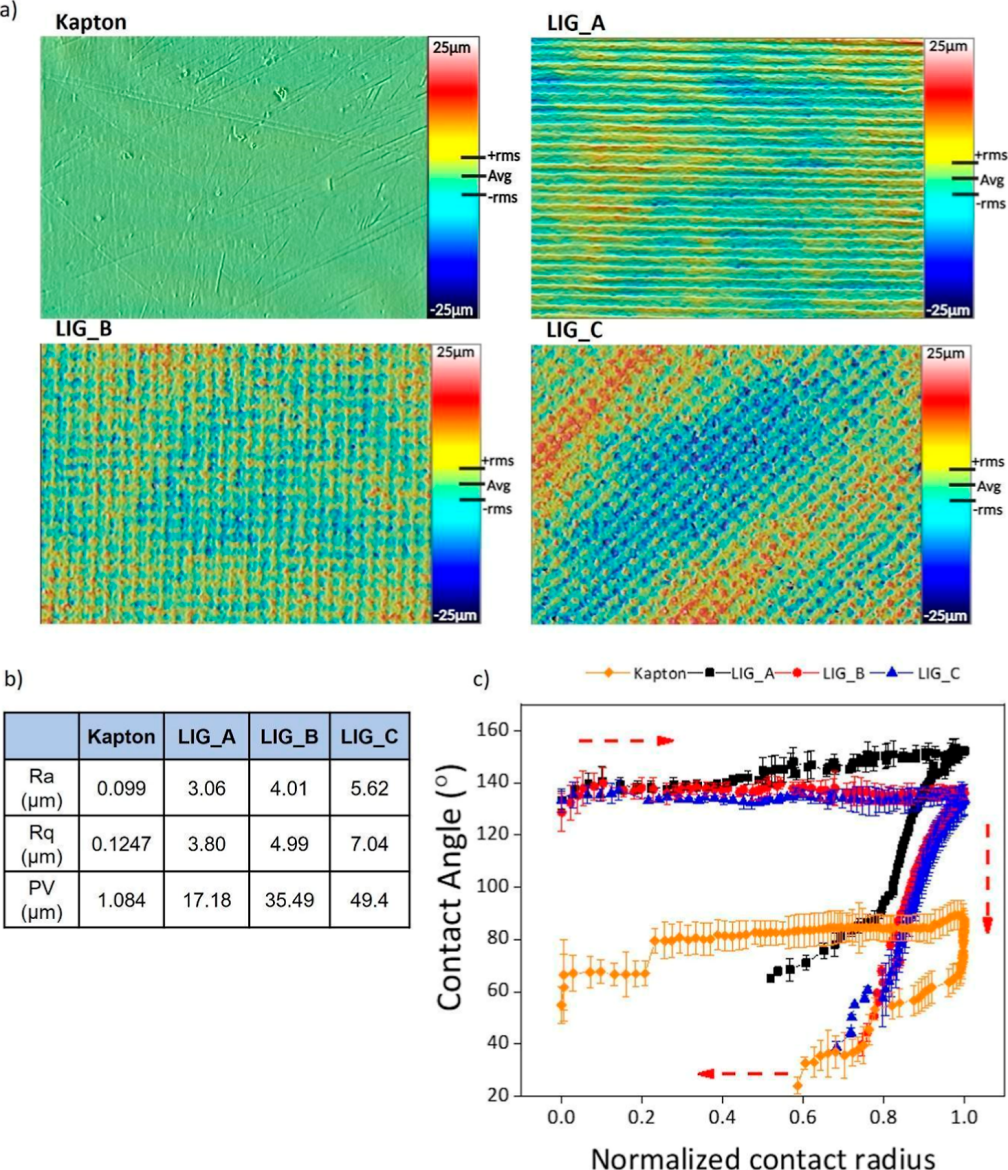
Surface characterization of LIG substrates with distinct laser engraving patterns. (a) Confocal microscopy images of Kapton, LIG_A, LIG_B, and LIG_C substrates, highlighting the unique surface roughness associated with each pattern. (b) Quantitative surface roughness metrics, including mean roughness (Ra), root-mean-square roughness (Rq), and peak–valley (PV) values for each engraving pattern. (c) Advancing and receding contact angle measurements of LIG substrates, illustrating wettability variations influenced by the laser-engraved patterns, where red arrows indicate the sequence in which the experiment was conducted.

**Figure 3:**
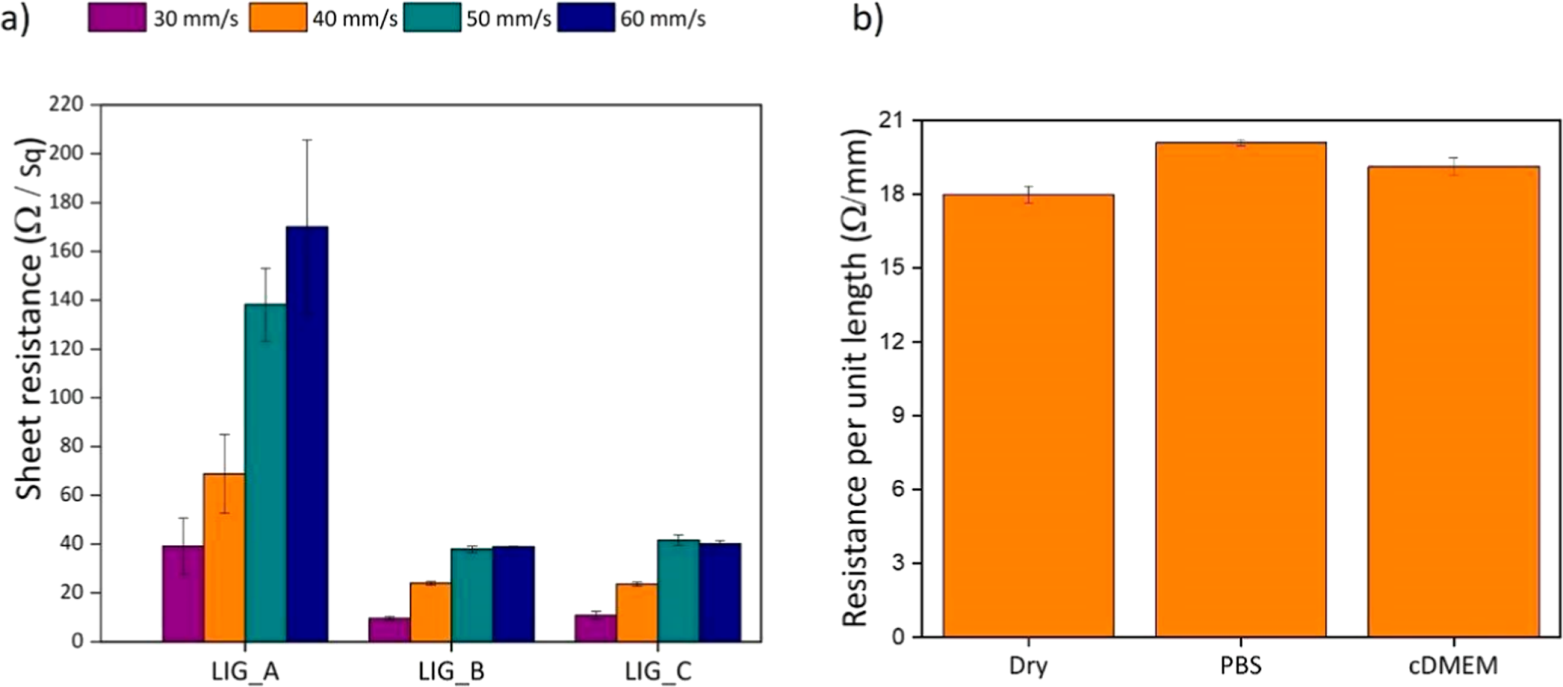
Electrical characterization of LIG substrates. (a) Shear resistance values as a function of pattern and laser etching speed at a fixed power of *P* = 1 W, highlighting the influence of these parameters on substrate stability. (b) Resistance per unit length (Ω/mm) measured in LIG substrate with different biological media, demonstrating the LIG response and electrical performance under various biological conditions. Data represent mean ± standard deviation of *N* = 30 replicates from minimum five independent substrates.

**Figure 4:**
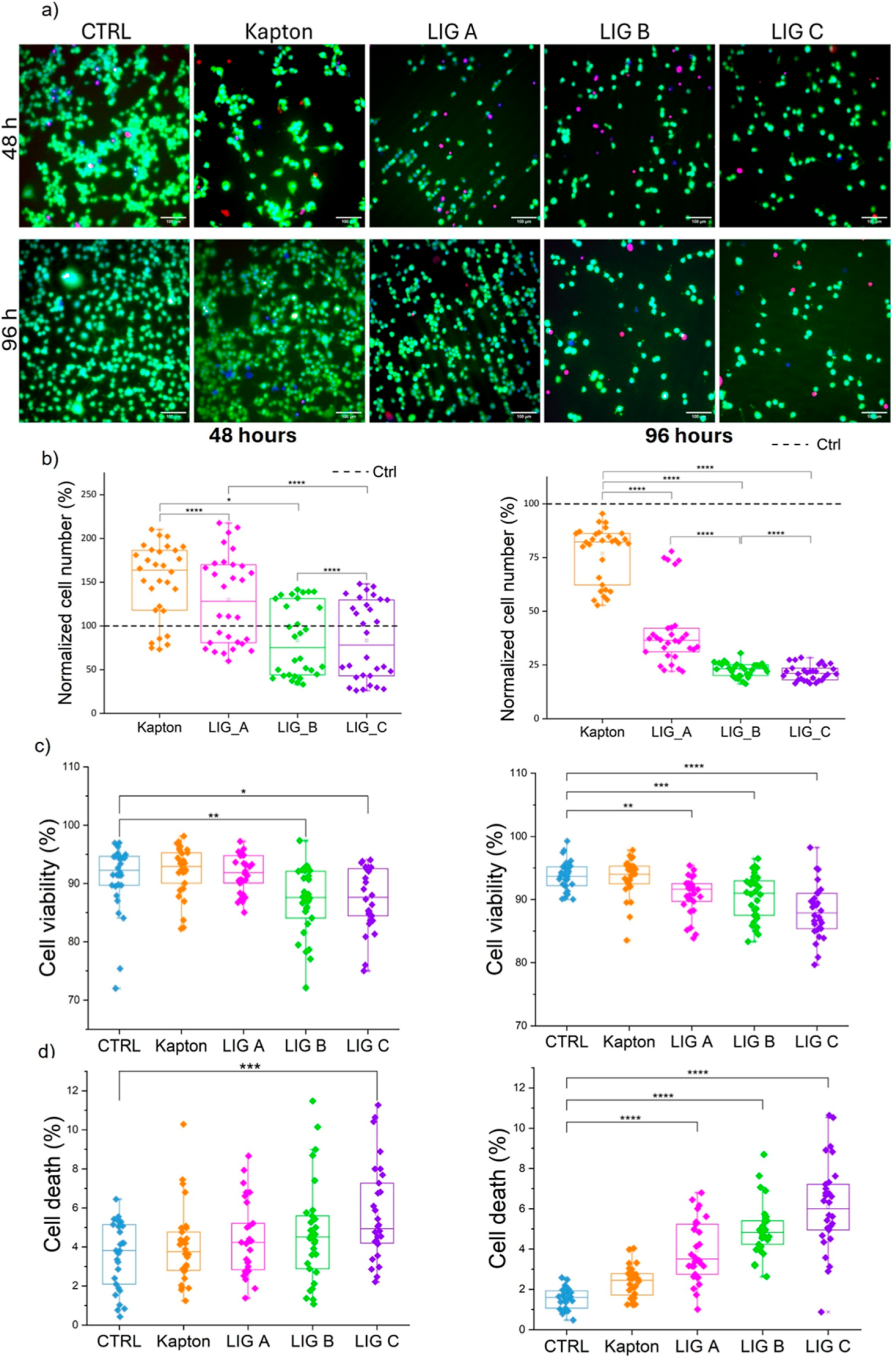
Fluorescence imaging and quantitative analysis of N2a cell density, viability, and death on laser-induced graphene (LIG) substrates. (a) Representative fluorescence micrographs of N2a cells cultured for 48 and 96 h on different substrates: glass (control), Kapton, and LIG patterns (LIG A, LIG B, and LIG C). Cell nuclei were stained with Hoechst (blue), viable cells with FDA (green), and dead cells with PI (red). Scale bar: 100 μm. (b–d) Quantitative analysis of cell density (b), cell viability (c), and cell death (d) at 48 and 96 h, highlighting cell–substrate interactions and the influence of surface architecture on cellular behavior. Data are presented as mean ± standard deviation (SD) from *N* = 30 replicates per condition. Statistical significance for cell density was assessed using one-way ANOVA followed by Tukey’s post hoc test for multiple pairwise comparisons among all groups. For cell viability and cell death, one-way ANOVA followed by Dunnett’s post hoc test was applied, using the control group as the reference. Significance levels: *p* < 0.05 (*), *p* < 0.01 (**), *p* < 0.001 (***), *p* < 0.0001 (****).

### Chemical Characterization

The transformation of Kapton polyimide into LIG is visually and structurally evident, as illustrated in Figure 1a. Through laser ablation at a wavelength of 532 nm, the carbon-rich precursor undergoes a precise reconfiguration, resulting in the formation of graphene-like structures.^38^ This outcome aligns with previous studies that highlight the efficiency of laser-induced graphitization in generating porous graphene architectures.^39,40^

Raman spectroscopy, shown in Figure 1b, further corroborates the structural transformation. The presence of a prominent G peak at ∼1580 cm^–1^ confirms the formation of sp^2^-hybridized carbon atoms, which is characteristic of graphitic materials. Simultaneously, the D peak at ∼1350 cm^–1^ exhibits slightly higher intensity than the G peak, suggesting a high density of sp^2^ structural defects, which is expected in LIG due to its laser-induced formation process. This level of structural disorder is advantageous for potential chemical functionalization and enhanced biocompatibility. Additionally, the emergence a broadened and less intense 2D peak at ∼2700 cm^–1^, a key indicator of layer thickness, suggests the formation of multilayer graphene with significant interlayer interactions. This spectral characteristic (I_2_D/I_G < 1) confirms that the obtained material is not pristine monolayer graphene but rather a structure with multiple stacked layers consistent with previous reports on the laser-induced carbonization of polyimide substrates.^41^ In contrast to pristine monolayer graphene, which typically displays a sharp, symmetric 2D band with I_2_D/I_G > 2 and a weak D band, the spectrum obtained here shows I_2_D/I_G < 1 and a pronounced D peak, confirming that the material is not pristine monolayer graphene but a defective multilayer graphene structure, in agreement with previous reports on the laser-induced carbonization of polyimide substrates.^42^ X-ray Photoelectron Spectroscopy (XPS) analysis, depicted in Figure 1c provides further insight into the chemical modifications induced by the laser treatment. The C_1s_ spectrum is dominated by a peak at 284.4 eV, assigned to C–C/C=C bonds typical of graphitic carbon, confirming the successful graphitization of the Kapton surface. Smaller components at higher binding energies are associated with C–N and oxygen-containing species (C–O, C=O and O–C=O), indicating that a low fraction of polar functional groups remains on the LIG surface.^43^ The quantitative XPS data in Figure 1d confirm the chemical modification induced by laser treatment, showing a rise in carbon content from 78.26% in raw Kapton to 97.04% in LIG, while oxygen decreases from 15.52% to 2.88%. This shift in elemental composition is reflected in the C/O ratio, which increases from 5.04 to 33.69, highlighting the efficient removal of most oxygen-containing functionalities. The reduction in nitrogen content suggests that C–N bonds are broken during the laser ablation process, likely resulting in the release of nitrogen-containing gases such as N_2_ or NOx. The resulting carbon-rich material, with its high conductivity and tunable surface chemistry, holds great promise for applications in flexible electronics, biosensing, and neural interfacing.

### Surface Roughness and Wettability: Enabling Biological Compatibility through Surface Engineering

The surface morphology and wettability of the LIG substrates were analyzed to determine whether laser-induced structural modifications can render the substrates suitable for biological applications. As shown in Figure 2a, the Kapton polyimide was laser-patterned into three distinct surface designs: LIG_A, LIG_B, and LIG_C, each resulting in a unique surface topography. 3D white light confocal microscopy revealed a progressive increase in surface roughness from untreated Kapton to the LIG substrates. While the raw Kapton exhibited a smooth and featureless surface, the laser-treated LIG patterns showed increasingly complex textures. LIG_A featured parallel ridges, LIG_B displayed a regular grid, and LIG_C presented an irregular, high-relief morphology. Both LIG_B and LIG_C were generated using two laser passes, but while LIG_B was produced by applying the second pass orthogonally to the first one, LIG_C was obtained with the second pass at an oblique angle, creating a dense array of rhomboidal features and a higher density of line intersections. This oblique cross-hatching increases the overlap between laser tracks and the local energy input at the crossing points, leading to more pronounced ablation and deeper valleys. These morphological differences are quantified in Figure 2b, where LIG_C displayed the highest roughness (Ra = 5.62 μm; PV = 49.4 μm), confirming a significantly enhanced surface texture. To evaluate whether these topographical changes modulate the wetting behavior and liquid adhesion, we measured advancing and receding contact angles as indicators of liquid affinity and surface adhesion. Contact angle hysteresis measurements are shown in Figure S2, demonstrating that the laser engraving pattern transforms Kapton into a high adhesion wetting state, with much stronger liquid pinning on LIG surfaces than on the pristine polymer. The results of Figure 2c show that the wetting regime changes markedly in all LIG substrates, with advancing contact angles θ_A_ ≈ 146° of LIG_A, θ_A_ ≈ 136.8° for LIG_B and θ_A_ ≈ 134° for LIG_C, compared to θ_A_ ≈ 82° for Kapton. Representative images of the advancing and receding water droplets on Kapton and on the three LIG patterns are provided in Figure S2 to visually illustrate these differences in wetting behavior. The receding angles are significantly reduced in the LIG patterns, with θ_R_ ≈ 72.5° for LIG_A and ≈47.7° for LIG_B, while the receding angle for LIG_C could not be reliably determined because the contactline remained pinned during volume reduction: Kapton exhibits θ_R_ ≈ 56.3°. This corresponds hysteresis values of *H* ≈ 73.5° and 84° for LIG_A and LIG_B, respectively, and an extremely high hysteresis regime for LIG_C, compared to *H* ≈ 25.8° for Kapton. For comparison, previous work reported that pristine CVD graphene supported on SiO_2_/Si and PET displays static water contact angles of about 74° and 84°, respectively, with very low contact angle hysteresis, i.e., a rather hydrophobic and weakly adhesive interface.^45^ In contrast, our LIG substrates combine high advancing contact angles with very low or even nonmeasurable receding angles and pronounced hysteresis, indicating strong liquid pinning and enhanced liquid adhesion compared to pristine graphene and related graphene-based conductive substrates. For rough and chemically heterogeneous surfaces, it is well established that the receding contact angle (θ_R_) and the contact angle hysteresis (*H* = θ_A_ – θ_R_) are more sensitive indicators of liquid–solid adhesion than the advancing angle alone; high θ_A_ can coexist with strong liquid adhesion when θ_R_ is significantly reduced, leading to large hysteresis and a high-adhesion wetting state rather than an easy-sliding, low-adhesion interface (as widely discussed in the wetting literature).^46–48^ In this sense, although the large θ_A_ values place LIG in the hydrophobic regime from a purely static perspective, the large H observed here reveals a high-adhesion wetting state with strong liquid retention, which is the relevant feature for maintaining stable hydrated and protein-coated interfaces in biological environments. This finding underscores the importance of controlling not only static contact angles but also contact angle hysteresis and liquid adhesion as key design parameters in the development of functional graphene-based biomaterials.^44–46^ The ability to modulate the wetting and adhesion regime of LIG through surface texturing opens new possibilities for biomedical interfaces, microfluidics and flexible electronic applications.

In addition to the effect of micro- and nanoscale roughness, the presence of a small fraction of oxygen-containing groups on the LIG surface, as revealed by XPS (Figure 1c,d), is expected to contribute to the unconventional wetting behavior observed here. The presence of oxygen-containing functional groups such as C–O, C=O and O–C=O can promote liquid pinning and contact angle hysteresis, thereby enhancing liquid retention on an otherwise largely hydrophobic, graphitic surface These residual oxygenated sites are also relevant from a biological perspective, as they are likely to modulate the adsorption of serum proteins and, consequently, cell adhesion and spreading on LIG.^47^ Furthermore, hydroxyl, carbonyl and carboxyl groups provide convenient anchor points for future covalent functionalization with bioactive molecules (e.g., peptides or extracellular matrix proteins), offering an additional handle to tailor the biological performance of LIG-based interfaces for tissue engineering and bioelectronic applications.

### Controlling Electrical Conductivity of LIG Substrates

To determine the most suitable synthesis conditions, an extensive range of laser power and scanning speed combinations was systematically evaluated. This parametric optimization enabled the identification of a fabrication window that ensured not only stable electrical conductivity but also surface characteristics favorable for biological applications.

The electrical properties of the LIG substrates were analyzed to evaluate the influence of pattern design and laser processing speed on conductivity, as well as their stability in biological environments, as depicted in Figure S3. The sheet resistance values measured across different configurations remained below 200 Ω/sq, indicating good electrical conductivity (Figure 3a). Despite some variability among patterns, all LIG substrates exhibited low shear resistance, highlighting the effectiveness of the laser-induced process in generating conductive carbon networks. A clear trend was observed between shear resistance and laser speed, where increasing the scanning velocity resulted in higher resistance values across all patterns (LIG_A, LIG_B, and LIG_C). This effect is attributed to a reduction in laser energy per unit area, leading to less efficient graphitization and, consequently, higher electrical shear resistance. Among the tested patterns, LIG_A showed the highest variability in conductivity, suggesting its microstructure is more sensitive to processing fluctuations. In contrast, LIG_B and LIG_C exhibited more consistent and lower resistance values, indicating more uniform graphitic networks and better structural continuity in their conductive paths. These findings were crucial in optimizing synthesis parameters to achieve a balance between electrical performance and surface topography, ensuring suitability for biointerface engineering. On this basis, a single scanning speed (see Table 1) was selected as the working condition for the rest of the study, and only LIG substrates fabricated under these optimized parameters were used for the detailed physicochemical, mechanical and biological characterizations presented in Figures 1, 2, 3b and 4.

To evaluate the stability of LIG substrates in biological conditions, we selected the LIG_A configuration, which exhibited the highest sheet resistance among the three designs, as a conservative worst-case scenario for electrical performance. The resistance per unit length (Ω/mm) of LIG_A was then measured in situ in dry conditions, phosphate-buffered saline (PBS), and Dulbecco’s Modified Eagle Medium (DMEM), supplemented with 10% (v/v) fetal bovine serum (FBS) and 1% penicillin–streptomycin (PS) solution (complete DMEM, cDMEM), the complete medium used for in vitro cell culture (Figure 3b). To account for minor variations in the distance between the manually placed electrodes, the resistance values are reported as resistance per unit length (Ω/mm). The results indicate that only minor variations in resistance per unit length were observed across the three media, indicating that LIG_A retains its electrical properties under all tested conditions. To further assess long-term stability, we performed an additional experiment in which LIG_A, LIG_B and LIG_C substrates were incubated in cDMEM for 7 days under standard cell culture conditions; after rinsing and drying, their sheet resistance was measured again (see Supporting Information Section and Figure S3). All LIG patterns showed an increase in sheet resistance after 7 days in cDMEM, consistent with partial surface modification, but the final values remained well below 200 Ω/sq, indicating that the substrates preserve a high level of conductivity after week-long exposure to complete culture medium. This is a significant finding, as maintaining conductivity in liquid environments is essential for biomedical applications, where physiological conditions are humid and rich in biomolecules, as represented here by cDMEM. Unlike other conductive materials that may experience performance degradation under high-humidity conditions,^35^ LIG demonstrates exceptional stability, positioning it as a promising candidate for next-generation implantable electronic devices. In addition, LIG-based electrodes have been reported to withstand extensive cyclic mechanical loading in flexible device configurations, further supporting their suitability for bioelectronic applications.^48,49^

Additionally, LIG presents distinct advantages over conventional graphene synthesized via chemical vapor deposition (CVD). The laser-induced process is not only simpler and more cost-effective but also environmentally friendly, avoiding the complex and resource-intensive fabrication steps associated with CVD-based methods.^50,51^ These findings reinforce the potential of LIG as an innovative material for bioelectronic interfaces and flexible conductive platforms.

### Mechanical Properties of LIG Substrates

Tensile stress–strain curves for Kapton and LIG-patterned samples are shown in Supporting Information Figure S4. All curves exhibit an initial linear regime at low strain, followed by a progressive deviation from linearity and saturation of the tensile stress until fracture. From these curves, the Young’s modulus was obtained as the slope of the best least-squares fit to the linear region of the stress–strain response, while the breaking stress and breaking strain were taken as the values of these quantities at sample failure. The corresponding mechanical parameters for Kapton and the different LIG samples are summarized in Table 2. No significant differences were observed in the Young’s modulus among the various LIG samples, whereas this quantity was significantly higher for the untreated Kapton films. Accordingly, the breaking stress and breaking strain were also larger for Kapton than for the laser-treated LIG samples, indicating a weakening of the polyimide after laser processing, although the different LIG treatments resulted in a similar degree of weakening. Furthermore, the Young’s modulus, breaking stress and breaking strain obtained for Kapton are consistent with values reported by the manufacturer and with mechanical property data available for thermoset polyimides.^52,53^

**Table 2:** Mechanical Properties of Kapton and LIG Substrates^a^

sample type	Young’s modulus (GPa)	breaking stress (MPa)	breaking strain (%)
LIG_A	2.028 ± 0.071	76.5 ± 1.7	30.4 ± 4.0
LIG_B	2.016 ± 0.057	77.2 ± 1.8	40 ± 11
LIG_C	1.966 ± 0.030	73.4 ± 1.1	35.6 ± 8.1
Kapton	2.644 ± 0.044	101.1 ± 3.2	57 ± 18

a Young’s modulus, breaking stress and breaking strain (mean ± standard deviation, *N* ≥ 5) for the different laser-induced graphene (LIG) patterns (LIG_A, LIG_B and LIG_C) and untreated Kapton films.

### LIG Substrates Biointeraction with Cells

The substrates obtained by LIG demonstrated excellent cell adhesion and growth without the need for additional surface treatments, thus suggesting a high level of biocompatibility. This finding highlights the suitability of LIG for potential future biomedical applications, showing its intrinsic ability to support cell growth without prior chemical and/or physical modifications.^54^ In this work, the use of different surface pattern designs aimed at optimizing cell–substrate interactions was also preliminarily explored. Moreover, we tested two different cell lines: human glioblastoma cells (U87-MG) and an immortalized mouse neuroblastoma cell line widely used in neuroscience research for neurotoxicity studies, N2a cells. Briefly, cells were directly seeded and grown onto the LIG substrates for 48 and 96 h, then samples were fixed and stained for fluorescence imaging acquisition. The results underscore the critical role of surface topography in cell maturation, orientation and proliferation (Figures 4a and S5a). The precise design of engraving patterns directly influences how cells interact with the substrate, promoting specific responses such as adhesion, migration, and cellular alignment.^55^ In Figures 4a and S5a–S6, representative fluorescence microscopy images show N2A cells and U-87 MG cell cultures, respectively, grown on different substrates after 48 and 96 h. The images highlight the influence of surface roughness on cellular growth, demonstrating how variations in substrate topography, determined by specific design patterns, modulate cell proliferation and distribution. Line patterns serve as physical guides that facilitate cell organization and alignment through the phenomenon of contact guidance.^56^ This mechanism enables cells to leverage the topographical features of the substrate for orientation and effective surface interactions. It was observed that in LIG_A, cells tend to distribute in a more orderly and efficient manner, as the line patterns facilitate the formation of focal adhesion points, consistent with previous studies.^56,57^ In contrast, LIG_B and LIG_C, featuring square and diamond patterns, exhibit a more complex topography that can act as a physical barrier to cell adhesion and migration. These patterns create fewer encouraging conditions for cell proliferation and organization due to a combination of reduced contact guidance and limitations in cell–surface interactions. Specifically, for U-87 MG, cell density was quantified over time (48 and 96 h), revealing that after 48 h (Figure S5b), the highest cell density was observed on the LIG_A substrate. This indicates that the surface properties of LIG_A are particularly positive for promoting initial cell adhesion and proliferation. Moreover, at 96 h (Figure S5c), the cell density on the LIG_A substrate exceeded that of the control, suggesting that this specific pattern could also perform comparably to the standard control in supporting sustained cell growth and proliferation.

## Biocompatibility Assessment of LIG Substrates

Fluorescence-based viability assays were performed with N2a cells (Figure 4) and revealed that LIG substrates support robust cell attachment and survival, confirming their overall cytocompatibility and in line with previous studies on LIG cytocompatibility and neural integration.^58–60^ At 48 h, cell density on all LIG patterns (LIG_A, LIG_B and LIG_C) was comparable to the glass control, indicating that the micro/nanostructured LIG surface supports early cell adhesion and proliferation (Figure 4b). This behavior is consistent with reports showing that hierarchical roughness and moderate surface energy in LIG promote early cell attachment by facilitating protein adsorption and focal adhesion formation.^59,61^

Notably, untreated Kapton, the precursor polymer prior to laser conversion, also sustained relatively high cell density at this early time point, consistent with its known chemical inertness and moderate hydrophilicity^62^ (Figure 4b). However, by 96 h clear differences emerged among substrates. While the control maintained high cell density, Kapton exhibited a marked decrease in cell number, suggesting poor long-term support for cell proliferation (Figure 4b). This reduction is likely related to its smooth and chemically inert surface, which lacks the topographical and chemical cues necessary for sustained adhesion and propagation during prolonged culture, as commonly observed for smooth polymeric substrates.^63^ In contrast, although LIG_A, LIG_B and LIG_C showed a decrease in cell density relative to the control at 96 h, their cell numbers remained substantially higher than those observed on Kapton (Figure 4b). This indicates that the micro/nanostructured LIG surfaces, even with pattern-dependent differences, provide a more favorable long-term microenvironment for cell retention and growth than the pristine polymer. Such behavior is consistent with prior evidence that graphene-based materials with micro/nanoroughness and oxygen-containing functionalities support improved protein adsorption and cell spreading relative to their polymeric precursors.^60,63^

Regarding cell viability, fluorescein diacetate (FDA) and propidium iodide (PI) quantification revealed statistically significant effects associated with the different LIG patterns (Figure 4c,d). At 48 h, viability in LIG_B and LIG_C was significantly lower than in the control, suggesting that specific pattern geometries or roughness levels may transiently influence cell adaptation during the early culture phase (Figure 4c). This tendency was also reflected in cell death (Figure 4d): LIG_C displayed a significantly higher percentage of PI-positive cells compared to the control. These findings suggest that the more pronounced surface roughness or chemistry associated with LIG_C induces an initial stress response, consistent with studies linking nanoscale sharp features or high-defect graphene surfaces to transient early cytoskeletal stress.^64,65^ By 96 h, viability differences became more pronounced: all LIG substrates (LIG_A, LIG_B and LIG_C) exhibited significantly lower viability compared to the control (Figure 4c), accompanied by higher levels of PI-positive cells (Figure 4d). The increased cell death on LIG at 96 h may reflect (i) surface-induced alterations in proliferation dynamics, (ii) differences in protein adsorption and nutrient distribution across microstructured topographies, or (iii) culture-related factors such as local confluence or mechanical constraints on patterned substrates phenomena previously documented for graphene-based micro topographies.^63,66^ Importantly, despite these viability differences, cell density remained consistently higher on LIG than on Kapton, reinforcing that the laser-induced micro/nanostructure provides a more favorable long-term interface than the precursor polymer.

Overall, these results demonstrate that LIG substrates are cytocompatible and able to support cell growth, with early performance comparable to the control and long-term performance superior to Kapton. The distinct responses observed across LIG_A, LIG_B and LIG_C highlight the importance of surface design, as micro/nanopatterning and variations in surface energy can modulate cell attachment, proliferation and survival. These findings align with previous reports showing that the hierarchical roughness and residual oxygen functionalities of graphene-based materials strongly influence protein adsorption, cytoskeletal organization and cell–substrate interactions.^61,62,67^ In this context, LIG offers an attractive platform for neural and bioelectronic interfaces, where fine control over surface structure is essential to tune cell behavior.^7,68^

## Conclusions

This study demonstrates the effectiveness of the laser-induced graphene (LIG) technique in producing highly customizable graphene-like substrates, with tunable electrical conductivity, roughness, and wettability achieved through precise laser parameter control (power, speed, frequency) and etching pattern design. Compared to conventional fabrication techniques, LIG offers a simplified, scalable, and cost-effective route to engineer surface properties that are essential for optimizing cell–material interactions.^26,69^ A critical finding of this work is the ability to modulate the wetting and adhesion behavior of the surface through laser patterning, inducing a high-adhesion wetting state with enhanced liquid retention on LIG patterns. This property is indispensable for the use of these substrates in biological environments, where cell culture media and biomolecules must interact efficiently with the material. While improvements in conductivity and morphology are necessary, they are insufficient if the material cannot sustain a stable hydrated interface; the combination of controlled roughness, contact angle hysteresis and liquid adhesion on the LIG substrates validates their practical usability for biomedical applications. Subsequently, preliminary biocompatibility assessments confirm that LIG substrates support cell adhesion and viability without requiring additional functionalization, underscoring their versatility in biomedical applications. The results also highlight the critical role of surface topography in regulating cell orientation and behavior. Variations in laser-induced etching patterns, such as linear, square, and rhomboidal designs, significantly influence cell proliferation and alignment, demonstrating that topographical cues can guide cell migration and organization. This ability to direct cellular growth is particularly relevant for neural tissue engineering, where controlled neuronal alignment and connectivity are essential for functional recovery. Of note, LIG substrates do not necessitate any additional surface treatment to promote cell adhesion and maturation, as it happens for other graphene-based supports that, especially for neural cell growth, must undergo a biological coating like poly lysine.^14^ In addition, tensile tests comparing untreated Kapton and LIG-patterned films show that laser processing induces only a moderate reduction in Young’s modulus, tensile strength and fracture strain, while preserving sufficient flexibility and mechanical robustness for handling, bending and deformation in flexible bioelectronic configurations. Overall, these findings reinforce the potential of LIG as a promising platform for tissue engineering and neuronal regeneration, where precise cell–material interactions are required for the formation of functional biological structures. By enabling simultaneous control over wettability (through a high-adhesion wetting regime), conductivity, topography and mechanical performance, LIG emerges as a powerful material for the development of next-generation biomedical devices, implantable systems and smart scaffolds.

## Supplementary Material



## Notes

In the Supporting Information file, we present: (i) a table displaying benchmarking of laser-induced graphene (LIG) developed in this work against recent graphene-based platforms (Table S1); (ii) a schematic representation of the experimental setup used to measure the electrical resistance of LIG substrates in biological media (Figure S1); (iii) contact angle measurements of LIG substrates with different surface patterns (Figure S2); (iv) the long-term electrical stability of LIG substrates in cell culture medium (Figure S3); (v) the stress–Strain curves and detail of its linear behavior at low strain values for patterns corresponding to LIG_A, LIG_B, LIG_C and the untreated Kapton samples (Figure S4); (vi) the visualization and quantification of U87-MG cellular interactions on LIG substrates (Figure S5); and (vii) the fluorescence microscopy images showing the interaction of U-87 MG cells cultured on different substrates (Figure S6) (PDF)
