# Supplementary material for: Laser-Induced Graphene Interfaces with Controlled Electrical Conductivity, Topography and Wettability for Biomedical Applications
Source: ACS Appl Nano Mater. 2025 Dec 16;8(51):24610–22. doi: 10.1021/acsanm.5c05398 (PMC12752773; doi:10.1021/acsanm.5c05398)
Supplement: Supplementary file 1 [file an5c05398_si_001.pdf]

Supporting Information

Laser-Induced Graphene Interfaces with Controlled Electrical Conductivity, Topography and Wettability for Biomedical Applications.

Lidia Lizbeth Hernández-Cubas[a,b,c], Paola Sánchez-Moreno[d], Andrea Capasso[e], Modesto T. Lopez-Lopez[d,f,g], Alejandro Moltó-Ramírez[d,f], Noel Rodríguez[b], Mattia Bramini †[a], and Carmen Lucía Moraila-Martínez †[b,c*]*

a) Universidad de Granada, Departamento de Biología Celular, E-18071 Granada, Spain

b) Universidad de Granada, Departamento de Electrónica y Tecnología de Computadores, E-18071 Granada, Spain

c) Universidad Autónoma de Sinaloa, Facultad de Biología, MX 80000 Culiacán Rosales Sinaloa, México

d) Universidad de Granada, Departamento de Física Aplicada, E-18071 Granada, Spain

e) International Iberian Nanotechnology Laboratory, Braga PT 4715-330, Portugal, Portugal

f) Instituto de Investigación Biosanitaria Ibs.GRANADA, E-18012 Granada, Spain

g) Universidad de Granada, Research Unit "Modeling Nature" (MNat), C.U. Fuentenueva, E-18071 Granada, Spain

* Corresponding authors: Mattia Bramini, mbramini@ugr.es; Carmen Lucía Moraila-Martínez, cmoraila@ugr.es

† These authors contributed equally

Table S1. Benchmarking of laser-induced graphene (LIG) developed in this work against recent graphene-based platforms (2023–2025), highlighting differences in sheet resistance, wettability, and cell-guidance performance. The comparison underscores the advantages of patterned LIG in achieving low sheet resistance, tunable wettability, and robust neuronal alignment relative to CVD graphene, graphene oxide hydrogels, and other bioelectronic interfaces reported in recent literature.

**Material / Technique (2024–2025)**	**Sheet Resistance (Ω/sq)**	**Wettability (Contact Angle Hysteresis)**	**Cell Guidance / Biocompatibility**	**Key Outcomes & Relevance**
Dallinger et al., 2023	Reported sheet resistance ranges (tunable)	Demonstrated continuous tuning of wettability (superhydrophilic to hydrophobic) and influence of surface chemistry	Biocompatibility discussion / surface chemistry effects	LIG with controlled atmosphere
Le et al., 2025. LIG for Biomedical Applications	10–100 Ω/sq	Not reported<br>Static contact angle: 80–110°	Suitable for neurons, fibroblasts; moderate alignment	Highlights multifunctionality and surface engineering potential
Lopes et al., 2024. CVD Graphene Neural Interfaces	300–1200 Ω/sq	Not reported<br>Static contact angle: 60–75°	Good neuronal adhesion; limited topographic control	High-quality but expensive; lacks surface patterning versatility
Li et al., 2025. Bioelectronic Graphene Platforms	150–2000 Ω/sq	Not reported<br>Static contact angle:<br>40–90°	Good general biocompatibility	Lower conductivity than LIG; requires post-processing
Du et al., 2023. GO Dimensionality & Cytoskeleton	Insulating / semiconductive	Not reported<br>Static contact angle:<br>40–60°	Affects cytoskeleton; alters migration & metastasis	Mechanistic insight; not conductive for electrodes
Zheng et al., 2024. GO Supramolecular Hydrogel	Not reported	Not reported<br>Static contact angle:<br><40°	Promotes wound healing; antibacterial; good biocompatibility	Chemical responsiveness; not suitable for electrodes
ChemElectroChem 2025. Graphene Sensors	20–200 Ω/sq	Not reported<br>Static contact angle:<br>90–115°	Not reported	Baseline graphene performance for sensing
This work. Patterned LIG (Kapton, CO_2 laser)	15–30 Ω/sq	Hysteresis contact angle (82–98°)	Strong alignment; viability >90%	High conductivity + pattern-controlled neuronal guidance

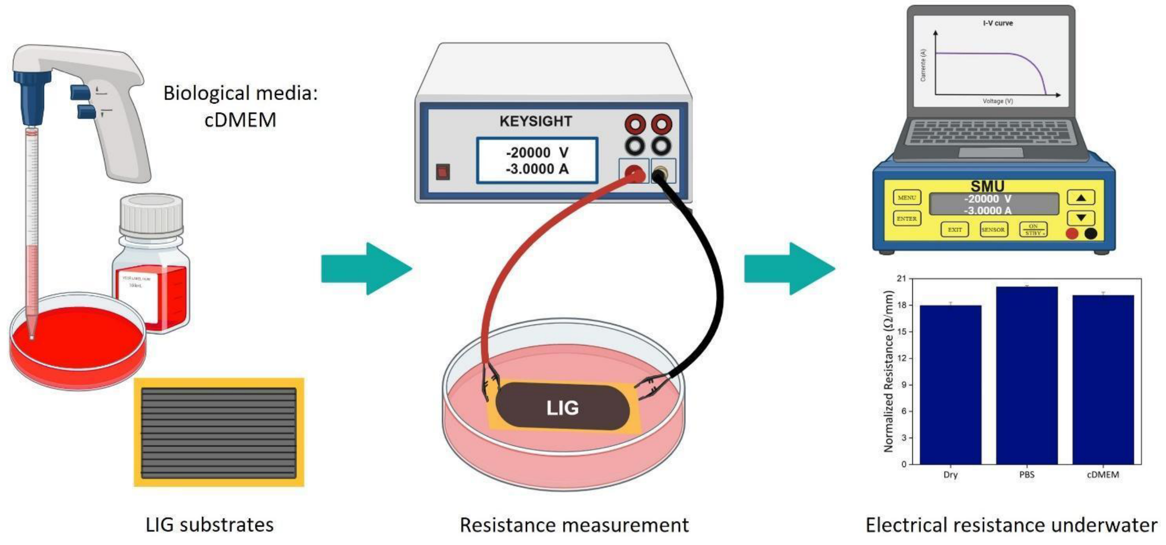

Figure S1. Electrical characterization set-up in biological media. Schematic representation of the experimental setup used to measure the electrical resistance of LIG substrates in biological media. The LIG substrates were designed with precise dimensions to fit within a Petri dish, allowing partial immersion in the biological medium while keeping the ends exposed for electrical connections. These exposed ends were linked to a Source Measurement Unit (SMU) to record current-voltage (I-V) curves. By applying a controlled voltage and measuring the resulting current, the electrical resistivity of the substrate in a biological environment was quantified, providing valuable insights into its conductivity and potential biomedical applications, particularly in understanding the electrical behavior of the substrates under these conditions.

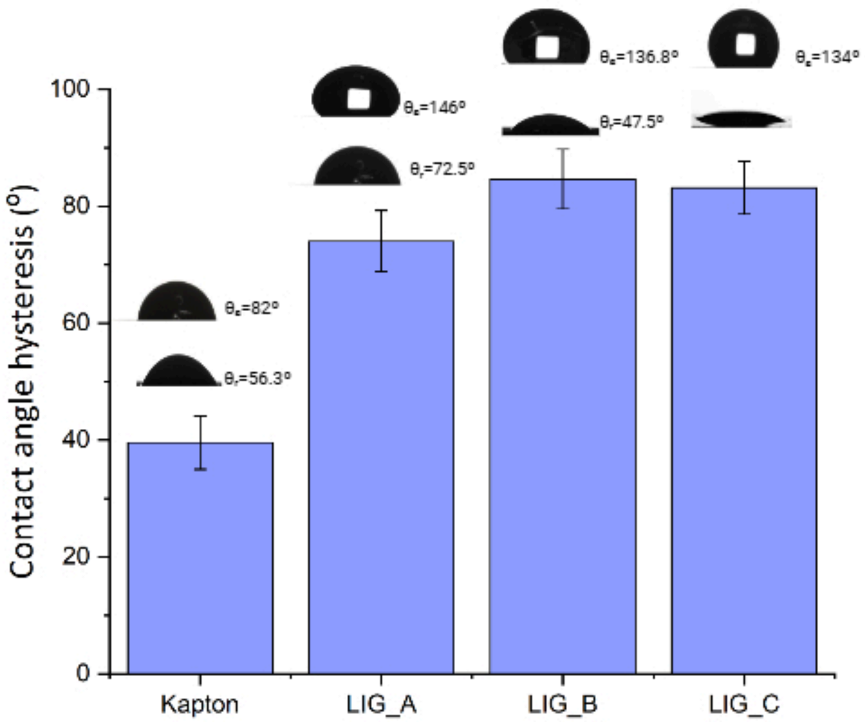

Figure S2. Contact angle measurements of LIG substrates with different surface patterns. Graphical representation of the contact angle measurements for each LIG substrate, illustrating how wettability properties vary as a function of the laser engraving pattern. Data represent mean ± standard deviation of minimum N=30 replicates from five independent substrates.

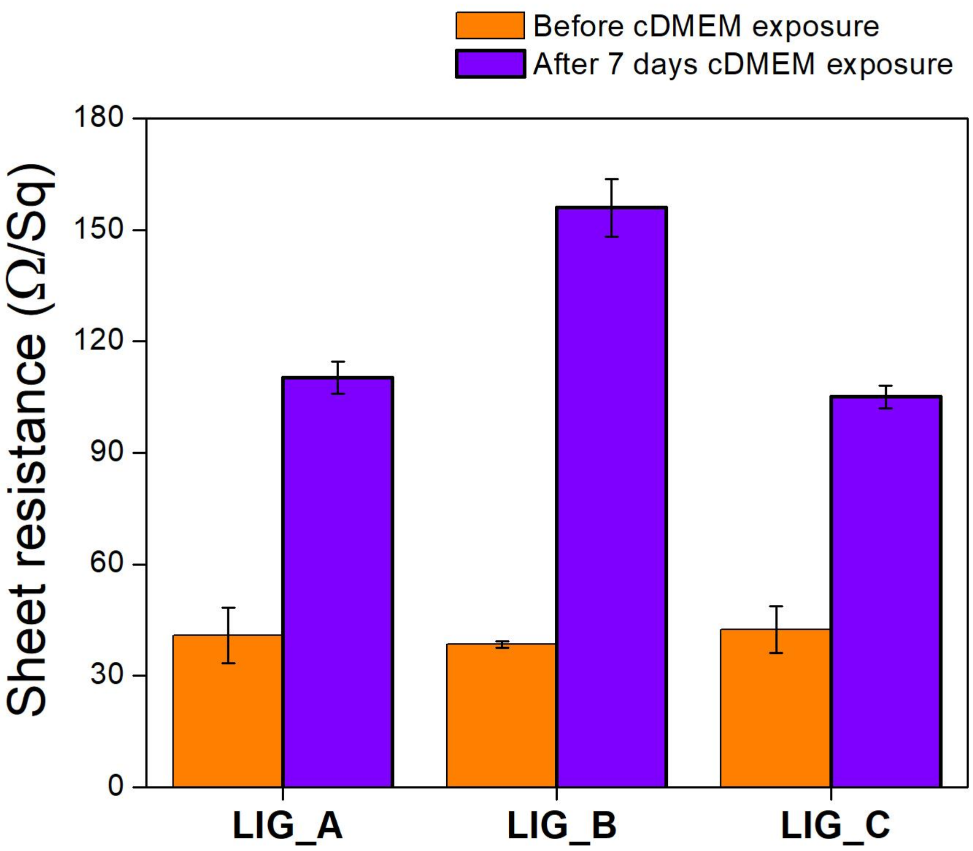

Figure S3. Long-term electrical stability of LIG substrates in cell culture medium. Sheet resistance of LIG substrates (LIG_A, LIG_B and LIG_C) measured in dry conditions (before cDMEM exposure) and after 7 days of incubation in complete DMEM (After 7 days cDMEM exposure) under standard cell culture conditions (37 °C, 5% CO_2). Data show an increase in sheet resistance after long-term exposure to cDMEM, while all values remain below 200 Ω/sq, confirming that the LIG patterns retain good electrical conductivity after 7 days in culture medium.

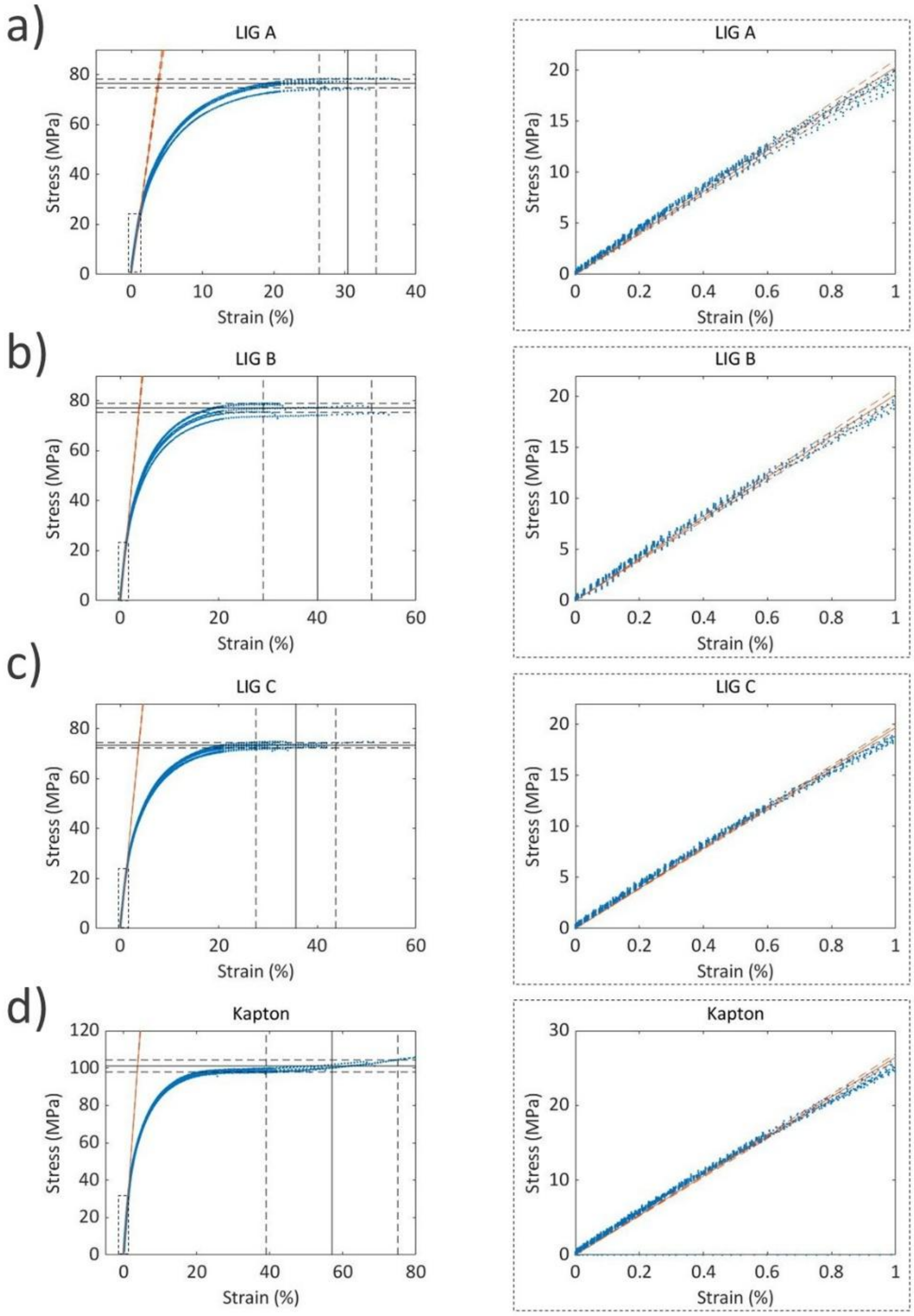

Figure S4. Stress-Strain curves and detail of its linear behaviour at low strain values for patterns corresponding to a) LIG A, b) LIG B, c) LIG C and d) for the untreated Kapton samples. The orange line represents the best linear fit for each sample with its standard deviation (orange dotted line) and the black lines represent the average values obtained for each breaking quantity with their standard deviation (black dotted line).

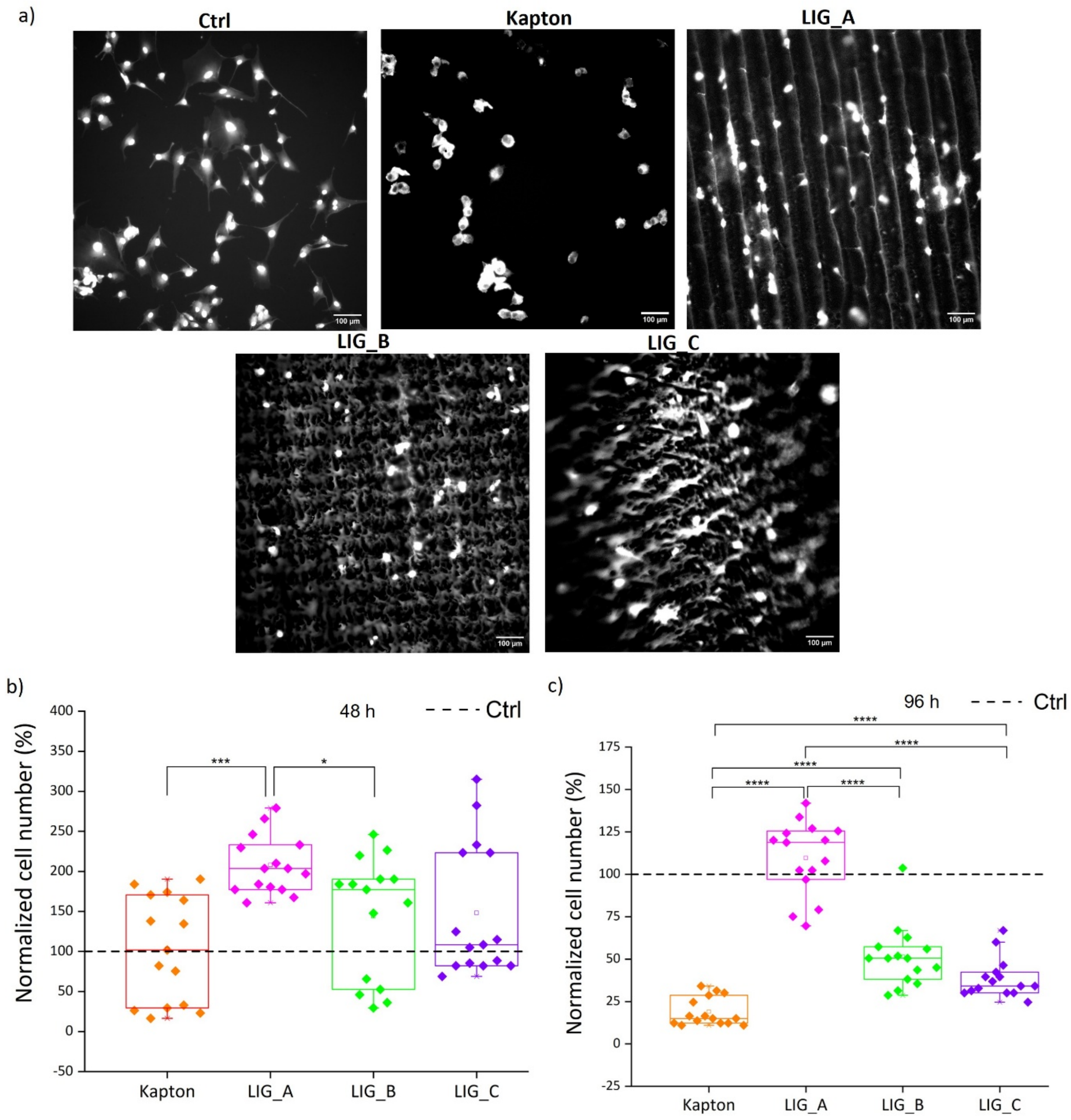

Figure S5. Visualization and quantification of cellular interactions on LIG substrates. a) Fluorescence microscopy images of U-87 MG cells cultured for 96 h on different substrates: Glass (control), Kapton, and LIG with patterns LIG_A, LIG_B and LIG_C. Scale bar: 100 µm. b) Quantitative analysis of cell density on the substrates at 48 and **c)** 96 hours, illustrating the influence of substrate composition and topography on cell proliferation and distribution. Data represent mean ± standard deviation of N=15 replicates from three independent biological experiments. Statistical significance was assessed using one-way ANOVA followed by Tukey's post hoc test for multiple pairwise comparisons among all groups. Significance levels: $p < 0.05$ (*), $p < 0.01$ (**), $p < 0.001$ (***), $p < 0.0001$(****).

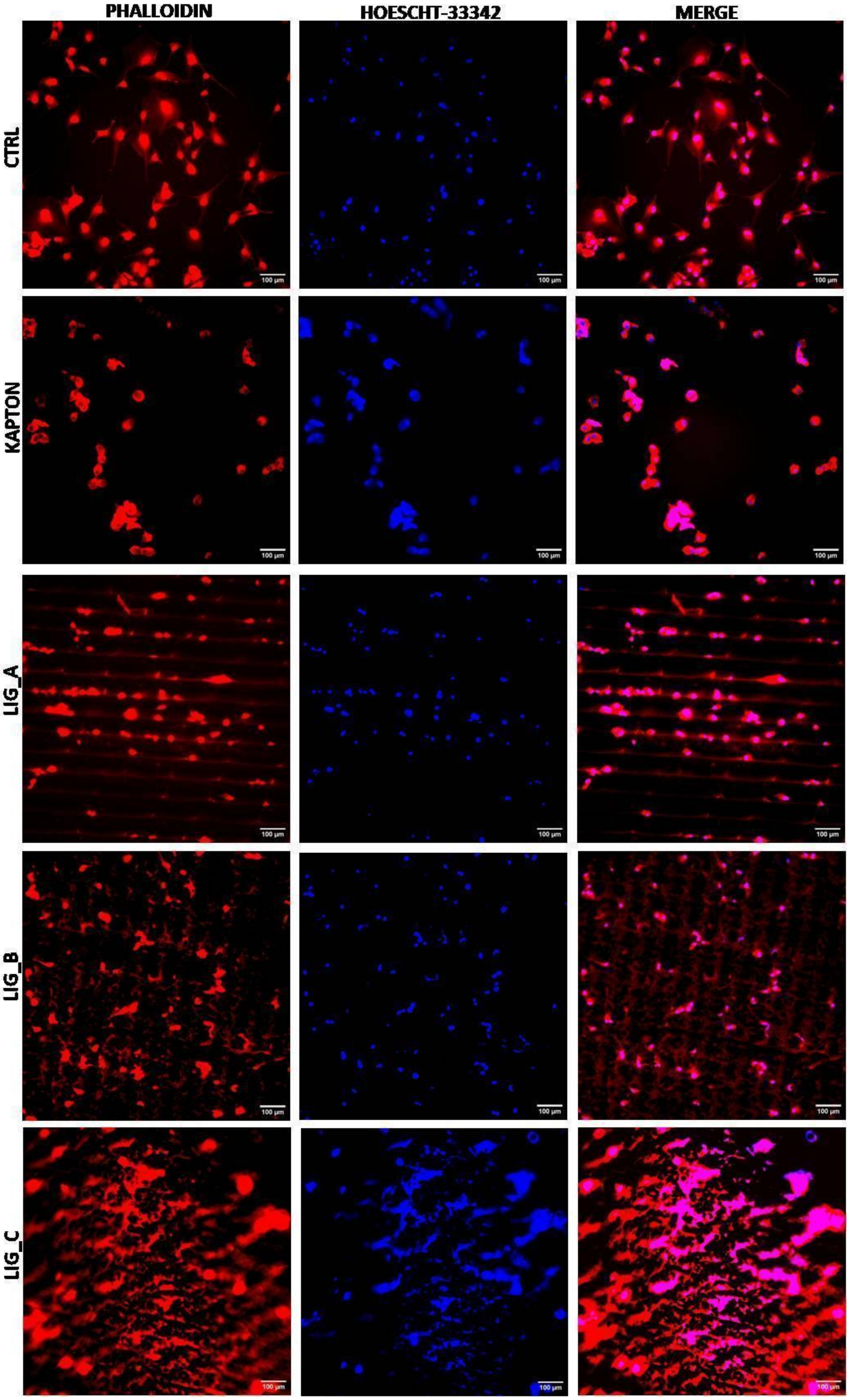
PHALLOIDIN
HOESCHT-33342
MERGE
CTRL
KAPTON
LIG_A
LIG_B
LIG_C
100 µm

Figure S6. Cell viability after 96 h of culture on LIG substrates. Fluorescence microscopy images showing the interaction of U-87 MG cells cultured on different substrates: control (glass), Kapton, and LIG with patterns A, B, and C. Cell viability were assessed using fluorescence microscopy. The panel presents representative images of U-87 MG cells stained with Hoechst 33342 (blue) for nuclear visualization and phalloidin (red) for actin cytoskeleton staining. (Scale bars: 100 μm).

REFERENCES

(1) Dallinger, A.; Steinwender, F.; Gritzner, M.; Greco, F. Different Roles of Surface Chemistry and Roughness of Laser-Induced Graphene: Implications for Tunable Wettability. *ACS Appl. Nano Mater.* **2023**, *6* (18), 16201–16211. https://doi.org/10.1021/acsanm.3c02066.

(2) Dinh Le, T.-S.; Tran, Y.-V.; Gao, Y.; Valerio, V. L.; Ge, Z.; Lim, C. T. Laser-Induced Graphene for Biomedical Applications: Innovations in Health Monitoring and Diagnostics. *Nanoscale Horiz.* **2025**, *10* (11), 2688–2721. https://doi.org/10.1039/D5NH00377F.

(3) Lopes, V.; Moreira, G.; Bramini, M.; Capasso, A. The Potential of Graphene Coatings as Neural Interfaces. *Nanoscale Horiz.* **2024**, *9* (3), 384–406. https://doi.org/10.1039/D3NH00461A.

(4) Li, J.; Yang, X. Advances in Bioelectronics for Neural Interfacing. *MRS Commun.* **2025**. https://doi.org/10.1557/s43579-025-00822-w.

(5) Du, Q.; Li, N.; Lian, J.; Guo, J.; Zhang, Y.; Zhang, F. Dimensional Effect of Graphene Nanostructures on Cytoskeleton-coupled Anti-tumor Metastasis. *Smart Medicine* **2023**, *2* (3), e20230014. https://doi.org/10.1002/SMMD.20230014.

(6) Zheng, C.; Wu, X.; Liu, M.; Lan, Y.; Liu, Q.; Cai, E.; Liao, Z.; Shen, J. Photothermal-enhanced in Situ Supramolecular Hydrogel Promotes Bacteria-infected Wound Healing in Diabetes. *Smart Medicine* **2024**, *3* (1), e20230047. https://doi.org/10.1002/SMMD.20230047.

(7) Jakubec, P.; Panáček, D.; Nalepa, M.; Rossetti, M.; Álvarez-Diduk, R.; Merkoçi, A.; Vasjari, M.; Kulla, L.; Otyepka, M. Graphene Derivatives as Efficient Transducing Materials for Covalent Immobilization of Biocomponents in Electrochemical Biosensors. *ChemElectroChem* **2025**, *12* (12), e202400660. https://doi.org/10.1002/celc.202400660.
